# Oral Health and Dental Management of Children With Down Syndrome: A Comprehensive Review

**DOI:** 10.7759/cureus.114489

**Published:** 2026-08-13

**Authors:** Abdullah S Alshamrani, Ghada Alshamrani, Abdulaziz Al-Shamrani, Mosleh Al-Shamrani

**Affiliations:** 1 Department of Pediatrics, Riyadh Armed Forces Hospital, Riyadh, SAU; 2 College of Dentistry, Riyadh Elm University, Riyadh, SAU; 3 Department of Pediatric Dentistry and Orthodontics, College of Dentistry, King Saud University, Riyadh, SAU

**Keywords:** anesthesia, dental, down syndrome, hypodontia, malocclusion, orofacial, osa

## Abstract

Down syndrome (DS) is the most common chromosomal disorder associated with intellectual disability and is marked by a wide array of systemic, craniofacial, and oral manifestations. Children with DS often present with dental anomalies and a range of medical comorbidities that significantly affect oral health and dental management. This review provides a comprehensive analysis of the dental, craniofacial, airway, and anesthetic challenges commonly encountered in children with DS, highlighting their practical implications for contemporary dental practice.

This narrative review synthesizes published studies, clinical guidelines, and review articles on oral health, craniofacial features, airway abnormalities, and perioperative considerations in children with DS. The synthesized evidence serves as a concise, practical resource for general dental practitioners.

Children with DS frequently exhibit delayed tooth eruption, hypodontia, abnormal tooth morphology, bruxism, periodontal disease, and malocclusion. Distinct craniofacial features, including relative macroglossia, midface hypoplasia, and a high-arched palate, pose substantial functional and orthodontic challenges. Obstructive sleep apnea (OSA) is common and can complicate airway management during sedation and general anesthesia. Additionally, conditions such as atlantoaxial instability (AAI) and congenital heart disease (CHD) require meticulous preoperative assessment and coordinated multidisciplinary care. Although dental caries rates may be similar to or lower than those in the general population, periodontal disease remains a principal source of oral morbidity.

Children with DS present with distinct oral, craniofacial, and medical characteristics that necessitate tailored dental care. Early implementation of preventive measures, ongoing dental monitoring, and robust collaboration among dental and medical professionals are essential for optimizing oral health, ensuring safe and effective treatment, and enhancing quality of life.

## Introduction and background

Down syndrome (DS), also known as trisomy 21, is the most common chromosomal disorder associated with intellectual disability. It results from the presence of an extra copy of chromosome 21 [1]. Individuals with DS typically present with a characteristic phenotype that includes developmental delay, hypotonia, distinctive craniofacial features, and a higher prevalence of congenital heart disease (CHD), endocrine disorders, hearing impairment, and other systemic conditions [2]. In Saudi Arabia, DS is notably prevalent, with an estimated incidence of one in 554 live births, a rate higher than that reported in many Western countries [3]. Advanced maternal age remains the principal risk factor, although demographic and cultural factors may also contribute to the increased prevalence observed in the Kingdom [1].

From a dental perspective, children with DS frequently exhibit oral, craniofacial, and airway abnormalities that significantly influence oral health and dental management [4-8]. Common findings include delayed tooth eruption, atypical tooth morphology, periodontal disease, malocclusion, hypodontia, relative macroglossia, and disturbances in craniofacial growth [5-8]. Medical comorbidities, such as CHD, obstructive sleep apnea (OSA), hypotonia, and possible cervical spine instability, may further complicate dental treatment planning, behavior management, and the safe delivery of sedation or general anesthesia [2,9]. Consequently, a thorough understanding of characteristic dental findings and associated medical conditions is essential for general dentists to provide safe, effective, and comprehensive oral healthcare for this population. Recent evidence emphasizes that periodontal disease is one of the most clinically important oral health concerns in individuals with DS. The increased susceptibility is thought to be multifactorial, involving immune dysregulation, altered oral microbiota, reduced manual dexterity, hypotonia, mouth breathing, and challenges in maintaining effective plaque control [10,11]. Although dental caries has traditionally been reported as less frequent in some children with DS, recent systematic reviews indicate that the evidence remains inconsistent and should not reduce the emphasis on caries prevention [4,12]. Therefore, preventive care should include early dental assessment, individualized oral hygiene instruction, caregiver education, dietary counseling, fluoride exposure, fissure sealants when indicated, and more frequent periodontal monitoring. Dental management of children with DS should be individualized and coordinated with the child’s medical team. Behavior guidance may require shorter appointments, desensitization visits, visual communication aids, positive reinforcement, and close caregiver involvement [2,13]. For children requiring sedation or general anesthesia, careful preoperative assessment is essential because airway obstruction, OSA, CHD, hypotonia, and possible cervical spine instability may increase perioperative risk [2,9]. Accordingly, dentists should document relevant medical history, obtain appropriate medical clearance when needed, and ensure that advanced procedures are performed in settings equipped for pediatric airway and medical emergencies.

## Review

Methods

This narrative review was conducted to provide a comprehensive overview of the oral health, craniofacial characteristics, airway abnormalities, and dental management considerations in children with DS. Relevant literature was identified through searches of major biomedical databases, including PubMed/Medical Literature Analysis and Retrieval System Online (MEDLINE), Scopus, Google Scholar, and Web of Science. Additional references were identified through manual screening of the reference lists of eligible publications. The search strategy included combinations of the following keywords and Medical Subject Headings (MeSH) where applicable: Down syndrome, trisomy 21, oral health, dental anomalies, periodontal disease, dental caries, malocclusion, craniofacial abnormalities, macroglossia, airway, obstructive sleep apnea, sedation, general anesthesia, orthodontics, pediatric dentistry, and special care dentistry. Original research articles, systematic reviews, meta-analyses, clinical guidelines, narrative reviews, and landmark publications relevant to pediatric patients with DS were considered. Priority was given to high-quality evidence, including recent systematic reviews, meta-analyses, clinical practice guidelines, and large observational studies published in English. Classic studies were included when they represented foundational evidence that continues to inform current clinical practice. The retrieved literature was reviewed and synthesized narratively. The evidence was organized into clinically relevant themes, including dental anomalies, periodontal disease, dental caries, craniofacial and soft tissue abnormalities, airway disorders, OSA, anesthesia and sedation considerations, CHD, and other oral manifestations. The objective was to provide a practical, evidence-based resource for dentists involved in the care of children with DS, emphasizing clinically relevant findings and current recommendations rather than performing a quantitative meta-analysis or formal assessment of study quality.

Dental and orofacial problems in children with DS

Children with DS often present with dental, craniofacial, and airway abnormalities that impact oral health, feeding, speech, and overall dental care. Early assessment and regular follow-up are crucial for prompt identification of issues and timely intervention. The principal dental and orofacial manifestations of DS and their practical implications are summarized in Table 1.

**Table 1:** Dental and orofacial manifestations of Down syndrome and practical implications for dental care

Manifestation	Typical presentation and clinical significance	Practical dental implications
Delayed tooth eruption	Delayed eruption of the primary and permanent dentitions; altered or asymmetric eruption sequence; retained primary teeth and unerupted teeth [14-20].	Perform clinical and radiographic surveillance, distinguish delayed eruption from agenesis, and coordinate preventive or interceptive orthodontic planning.
Hypodontia or oligodontia	Often involves third molars, maxillary lateral incisors, mandibular second premolars or incisors; may cause spacing, retained primary teeth, and occlusal problems [21-26].	Use panoramic assessment during the mixed dentition when clinically indicated; preserve useful primary teeth and plan space management or later prosthetic replacement.
Abnormal tooth size, shape, or structure	Microdontia, peg-shaped or conical crowns, taurodontism, enamel hypoplasia or hypocalcification, and short or altered roots [27-34].	Strengthen prevention, use minimally invasive restorative care, and evaluate crown and root morphology before orthodontic, restorative, or endodontic treatment.
Bruxism	Sleep or awake grinding and clenching may cause tooth wear, sensitivity, masticatory muscle or temporomandibular symptoms, and restoration failure [35-41].	Ask caregivers about grinding, document wear, restore damaged teeth when necessary, assess sleep-disordered breathing, and use splints only in selected cooperative older patients.
Periodontal disease	Early-onset gingivitis or periodontitis may progress rapidly to attachment loss, alveolar bone loss, mobility, and premature tooth loss [42-49].	Provide caregiver-assisted plaque control, professional prophylaxis, and risk-based shorter recalls; refer for periodontal care when inflammation or destruction persists.
Dental caries	Caries prevalence is variable. Delayed eruption and spacing may be protective, but poor hygiene, diet, mouth breathing, enamel defects, and access barriers can increase risk [50-56].	Use individual caries-risk assessment, fluoridated toothpaste and professional fluoride, dietary counseling, fissure sealants, and prompt restoration of cavitated lesions.
Relative macroglossia and orofacial hypotonia	Tongue protrusion, poor lip seal, drooling, and speech, feeding, or swallowing difficulties may occur and may worsen mouth breathing [57-64].	Support caregiver-assisted hygiene, screen for airway problems, and refer to speech-language, feeding, or otolaryngology services when functional problems are present.
Midface hypoplasia, narrow palate, and malocclusion	Maxillary deficiency, Class III tendency, anterior or posterior crossbite, open bite, crowding, or spacing are common [65-90].	Arrange early orthodontic review; undertake interceptive or comprehensive treatment only when cooperation, periodontal health, oral hygiene, and medical status are suitable.
Other oral and functional findings	Fissured tongue, lip fissuring or angular cheilitis, candidal carriage, drooling, dysphagia, and possible temporomandibular dysfunction may occur [91-136].	Reinforce tongue and lip hygiene, manage irritation or infection, screen for swallowing difficulty and pain, and refer when functional symptoms persist.

Delayed Tooth Eruption

Delayed tooth eruption is a hallmark oral finding in DS, affecting both the primary and permanent dentitions [14-18]. Children with DS often experience delayed eruption compared with typically developing children, and eruption may be delayed by several months to, in some cases, nearly two years [14-17]. In addition to delayed timing, the eruption sequence may be altered, and asymmetrical eruption patterns may be observed, particularly in the permanent dentition [15,16]. Recent large-scale Japanese survey data further support that DS is associated with delayed primary tooth eruption, persistence of primary teeth, delayed permanent tooth eruption, fewer erupted permanent teeth, and a higher likelihood of unerupted teeth or early tooth loss [17,18]. These eruption disturbances may be compounded by hypodontia and other developmental dental anomalies, which can further contribute to retained primary teeth, spacing, crowding, malocclusion, and increased orthodontic complexity [19]. Delayed eruption may also affect functional development, as recent evidence suggests an association between deciduous tooth eruption, physical development, motor milestones, and feeding adaptation in young children with DS [20]. Therefore, regular clinical and radiographic surveillance is recommended to monitor eruption progress, detect missing or impacted teeth early, guide preventive and orthodontic planning, and support feeding and oral function as part of multidisciplinary care.

Missing Teeth (Hypodontia)

Hypodontia, defined as the congenital absence of one or more teeth, is substantially more common in individuals with DS than in the general population [21,22]. It is one of the most frequently reported developmental dental anomalies in this group and may affect both the primary and permanent dentitions, although it is particularly well documented in the permanent dentition [21-24]. The teeth most commonly absent include the third molars, maxillary lateral incisors, mandibular second premolars, mandibular incisors, and maxillary second premolars [21,22]. A meta-analysis reported a high prevalence of permanent tooth agenesis in individuals with DS, with more severe and multiple-tooth involvement than is usually observed in nonsyndromic hypodontia [22]. More recent pattern-based analysis also suggests that tooth agenesis in DS may follow recognizable distribution patterns, which is clinically relevant for diagnosis, orthodontic planning, and long-term restorative decision-making [23].

The etiology of hypodontia in DS is likely multifactorial and related to altered odontogenesis associated with trisomy 21, including disturbances in dental lamina activity, tooth-bud initiation, and craniofacial development [21,24,25]. Clinically, missing teeth may contribute to spacing, retained primary teeth, delayed eruption of successors, altered occlusion, impaired mastication, speech difficulties, and compromised dental and facial esthetics [21,22,26]. In children with multiple missing teeth, the condition may further increase orthodontic complexity and require staged interdisciplinary management involving pediatric dentistry, orthodontics, prosthodontics, and, when appropriate, oral surgery [22,23,26]. Early diagnosis is important because treatment needs change with growth and dental development. Panoramic radiography is useful during the mixed dentition stage to confirm congenitally missing teeth, identify retained primary teeth, and distinguish true agenesis from delayed eruption [21,22]. Management should be individualized and may include monitoring, preservation of retained primary teeth when functional, space maintenance or closure, orthodontic alignment, adhesive prosthetic replacement, or definitive prosthetic rehabilitation after growth completion [23,26].

Abnormalities in Tooth Size, Shape, and Structure

Abnormalities in tooth size, shape, and structure are common in individuals with DS and form an important component of their distinctive dental phenotype [27-31]. Frequently reported findings include microdontia, peg-shaped or conical teeth, taurodontism, altered crown morphology, enamel hypoplasia, enamel hypocalcification, and, less commonly, root dilaceration or other root-form anomalies [27-31]. These abnormalities may affect both the primary and permanent dentitions, although most radiographic studies focus on the permanent dentition [27-30]. Microdontia and altered crown shape may compromise dental esthetics, spacing, occlusion, and masticatory efficiency, while enamel defects may increase plaque retention, tooth sensitivity, tooth wear, and susceptibility to caries when preventive care is inadequate [30,31].

Root and crown dimensions also have clinical relevance in treatment planning. Cone-beam computed tomography studies have shown that teeth in individuals with DS may be smaller than those of unaffected controls, with differences in crown and root measurements that should be considered before orthodontic or restorative treatment [32,33]. Short or morphologically altered roots, taurodontism, and reduced periodontal support may complicate orthodontic tooth movement, endodontic access, and long-term restorative prognosis [27,32,33]. Therefore, early clinical examination supported by appropriate radiographic assessment is essential to identify dental anomalies, assess caries and periodontal risk, and plan individualized preventive, restorative, orthodontic, and prosthetic care [31,34].

From a practical standpoint, management should focus on preserving tooth structure and minimizing treatment complexity. Preventive strategies include meticulous plaque control, fluoride therapy, dietary counseling, and regular monitoring of enamel defects and tooth wear. Esthetic or functional problems related to microdontia, peg-shaped teeth, or spacing may be managed with minimally invasive adhesive restorations, composite build-ups, or prosthetic replacement when indicated. Orthodontic treatment should be planned cautiously, with careful assessment of root morphology, periodontal status, oral hygiene ability, and patient cooperation before active tooth movement is initiated [31-34].

Bruxism (Tooth Grinding)

Bruxism, defined as repetitive masticatory muscle activity involving tooth grinding, clenching, bracing, or thrusting of the mandible, may occur during sleep or wakefulness [35,36]. It is a common oral parafunctional behavior in individuals with DS and appears to occur more frequently than in the general pediatric population [37,38]. The reported prevalence varies considerably across studies because of differences in age, diagnostic criteria, caregiver reporting, clinical examination, and the use of study casts or polysomnography [37,38]. Its etiology in DS is likely multifactorial and may involve craniofacial morphology, malocclusion, mouth breathing, generalized hypotonia, sleep-disordered breathing, behavioral factors, sensory processing differences, and impaired neuromuscular control [37,39,40].

Chronic bruxism can lead to clinically significant complications, including excessive occlusal tooth wear, enamel loss, dentin exposure, tooth sensitivity, reduced vertical dimension, masticatory muscle pain, temporomandibular joint discomfort, headaches, and fracture or failure of dental restorations [37-39]. In children with DS, diagnosis may be challenging because pain or discomfort may be under-reported, and clinical signs such as tooth wear must be interpreted carefully in relation to age, dentition stage, diet, and parafunctional history [37,39]. Therefore, dentists should actively ask caregivers about audible grinding, clenching during the day, sleep quality, snoring, mouth breathing, morning jaw fatigue, and behavioral triggers [37,40,41].

Management should be individualized and conservative, with the primary goals of protecting the dentition, reducing pain, improving function, and addressing contributing factors. Initial care includes caregiver education, monitoring of tooth wear, optimization of oral hygiene, restoration of damaged teeth when indicated, management of sharp enamel edges, and referral for assessment of sleep-disordered breathing when snoring, witnessed apnea, or persistent mouth breathing is present [37,40,41]. Occlusal splints may be considered in selected older children or adolescents, but their use in growing children requires caution because of eruption, craniofacial growth, cooperation, and airway considerations [39,41]. Multidisciplinary care involving pediatric dentistry, orthodontics, sleep medicine, otolaryngology, physiotherapy, and behavioral support may be required in persistent or severe cases [37,39-41].

Periodontal Disease

Periodontal disease is one of the most significant oral health problems in individuals with DS and represents an important cause of early tooth loss in this population [42,43]. Individuals with DS show increased susceptibility to gingival inflammation and periodontitis, with disease often beginning at a younger age and progressing more rapidly than in unaffected individuals [42-45]. This heightened vulnerability is multifactorial and is related to immune dysregulation, impaired host response, altered neutrophil chemotaxis and phagocytic activity, increased inflammatory mediator release, oral microbiome differences, mouth breathing, malocclusion, reduced manual dexterity, and difficulty maintaining effective plaque control [44-49].

Clinically, periodontal involvement may initially present as gingival erythema, bleeding on probing, edema, and plaque-induced gingivitis, but it may progress to periodontal pocketing, clinical attachment loss, alveolar bone resorption, tooth mobility, and premature tooth loss [42-45]. Importantly, periodontal destruction in DS cannot always be explained by plaque accumulation alone; several studies suggest that altered innate and adaptive immune responses play a major role in the severity and early onset of periodontal inflammation [44-49]. This explains why some individuals with DS may demonstrate disproportionate periodontal breakdown despite relatively modest plaque levels.

Management should begin early and focus on prevention, caregiver education, and regular periodontal monitoring. Preventive care should include individualized oral hygiene instruction, caregiver-assisted brushing, interdental cleaning when feasible, fluoride exposure, dietary counseling, professional prophylaxis, and frequent recall visits based on periodontal risk [42-45]. Children and adolescents with signs of persistent gingival inflammation, attachment loss, tooth mobility, or radiographic bone loss should be referred for periodontal assessment. Because conventional periodontal therapy may be less predictable in DS, long-term maintenance, reinforcement of home care, and coordination between pediatric dentists, periodontists, caregivers, and physicians are essential [44,45].

Dental Caries

Dental caries in individuals with DS has been reported as similar to or, in some studies, lower than that observed in the general pediatric population [50-52]. This apparently reduced caries experience is likely multifactorial and may be related to delayed tooth eruption, congenitally missing teeth, microdontia, wider interdental spacing, shallower fissures, and salivary factors such as increased buffering capacity and altered electrolyte composition [50,53,54]. These factors may reduce the duration of exposure to cariogenic challenges and facilitate natural cleansing in some patients.

However, dental caries remains an important clinical concern in children with DS and should not be underestimated [50-52,55]. Protective anatomical or salivary factors may be outweighed by poor oral hygiene, reduced manual dexterity, caregiver-dependent brushing, frequent intake of fermentable carbohydrates, feeding difficulties, xerostomia or mouth breathing, limited cooperation during oral care, and barriers to accessing preventive dental services [54-56]. Some regional studies, including data from Riyadh, have reported a high caries burden among individuals with DS, highlighting the importance of local context, diet, fluoride exposure, socioeconomic factors, and access to regular dental care [55]. Preventive management should therefore be individualized rather than based on the assumption that children with DS are naturally protected from caries. Regular dental examinations, caries-risk assessment, caregiver education, twice-daily fluoridated toothbrushing, dietary counseling, topical fluoride application, fissure sealants when indicated, and early restoration of cavitated lesions are essential components of care [50-56]. Close follow-up is particularly important in children with feeding difficulties, poor plaque control, enamel defects, reduced salivary flow, or limited access to dental services.

Craniofacial and soft tissue abnormalities

Macroglossia (Large Tongue Appearance)

Although macroglossia is frequently described as a characteristic feature of DS, true anatomical enlargement of the tongue is uncommon. In most individuals, the appearance of a large tongue is better explained as relative macroglossia, in which the tongue appears disproportionately large because of a relatively small oral cavity, midfacial hypoplasia, maxillary underdevelopment, hypotonia, and reduced neuromuscular control [57-59]. This craniofacial configuration commonly contributes to tongue protrusion, open-mouth posture, and difficulty maintaining an effective lip seal [58-60].

Altered tongue posture and reduced orofacial muscle tone may affect several functional domains, including articulation, speech intelligibility, feeding, swallowing, drooling control, and masticatory efficiency [60-63]. In addition, chronic tongue protrusion and mouth breathing may contribute to malocclusion patterns such as anterior open bite, posterior crossbite, high-arched palate, and Class III skeletal tendency, particularly when combined with maxillary hypoplasia and delayed craniofacial growth [58,59]. These changes may also worsen oral dryness, plaque accumulation, gingival inflammation, and functional oral hygiene difficulties.

Management should be individualized and multidisciplinary. Early assessment by pediatric dentists, orthodontists, speech-language therapists, otolaryngologists, and feeding specialists is important when tongue protrusion, poor lip seal, drooling, speech impairment, feeding difficulty, or sleep-disordered breathing is present [61-64]. Conservative approaches include orofacial myofunctional therapy, speech and feeding therapy, caregiver-directed oral-motor exercises, and management of nasal obstruction or OSA when suspected. Orthodontic palatal plate therapy may be considered in selected young children as part of combined orofacial regulation therapy, although available evidence suggests benefit mainly when used with physiotherapy, speech-language therapy, and close follow-up [64].

Midface Hypoplasia

Midface hypoplasia is a characteristic craniofacial feature of DS, typically presenting as a flat nasal bridge, reduced midfacial projection, maxillary underdevelopment, and relative mandibular prominence [65-68]. Cephalometric and three-dimensional imaging studies have demonstrated reduced maxillary dimensions, shorter cranial base measurements, and altered maxillomandibular relationships in individuals with DS compared with non-syndromic controls [65-70]. These skeletal changes contribute to the typical facial profile observed in DS and may become more evident with growth.

Insufficient maxillary development frequently contributes to a skeletal Class III pattern, in which the mandible appears prominent relative to the deficient midface rather than because of true mandibular prognathism alone [65,68-70]. Reduced transverse and sagittal maxillary growth may also contribute to a narrow maxillary arch, dental crowding, anterior crossbite, posterior crossbite, anterior open bite, and other malocclusion patterns commonly reported in children and adolescents with DS [69,71]. These occlusal problems may affect mastication, speech, oral hygiene access, esthetics, and the complexity of orthodontic treatment.

Midface hypoplasia may also influence airway function. Maxillary deficiency, relative macroglossia, generalized hypotonia, adenotonsillar hypertrophy, and craniofacial restriction can collectively contribute to chronic mouth breathing, upper-airway obstruction, and OSA [72,73]. Because symptoms alone may underestimate sleep-disordered breathing in DS, children with snoring, restless sleep, mouth breathing, daytime sleepiness, behavioral changes, or persistent open-mouth posture should be evaluated carefully and referred for sleep or otolaryngology assessment when indicated [72,73].

Management requires an individualized and multidisciplinary approach. Pediatric dentists and orthodontists should monitor maxillary growth, occlusion, arch width, eruption, and oral hygiene from an early age. Orthodontic treatment may include interceptive management of crossbite, maxillary expansion, facemask therapy, or comprehensive orthodontic care in selected patients, depending on age, cooperation, skeletal maturity, periodontal status, and medical risk [69-71]. Coordination with otolaryngologists, sleep specialists, speech-language therapists, and pediatricians is important when airway obstruction, speech difficulty, feeding problems, or significant malocclusion are present [72,73].

High-Arched and Narrow Palate

Children with DS frequently present with craniofacial abnormalities involving the maxilla and palate, including a narrow maxillary arch, high-arched or constricted palate, shelf-like or “stair” palate, and posterior crossbite [74-77]. These findings are mainly related to altered craniofacial growth, midface hypoplasia, maxillary underdevelopment, reduced oral cavity volume, and abnormal orofacial muscle tone [74-76]. Although palatal morphology in DS may vary with age, morphometric studies have shown that the hard palate and maxillary arch may be smaller, narrower, or morphologically altered compared with typically developing controls [74-76].

The resulting transverse maxillary deficiency can interfere with normal dental eruption, arch coordination, mastication, speech articulation, and oral hygiene access [75-77]. Posterior crossbite is particularly common and may be associated with maxillary constriction, altered tongue posture, and skeletal Class III tendency [77-79]. If not recognized early, these occlusal abnormalities may worsen with growth and contribute to functional imbalance, asymmetric mandibular positioning, tooth wear, periodontal stress, and increased orthodontic complexity [77-79]. Therefore, early orthodontic evaluation is recommended to assess arch width, palatal morphology, occlusion, eruption sequence, and the need for interceptive treatment, including maxillary expansion or other orthopedic approaches when clinically appropriate [78,79].

Management should be individualized according to age, cooperation, medical status, oral hygiene ability, airway concerns, and periodontal condition. Interceptive orthodontic treatment may improve transverse arch relationships and reduce functional crossbite, but treatment goals should remain realistic because children with DS may have reduced cooperation, increased periodontal susceptibility, and complex craniofacial growth patterns [78,79]. Close collaboration among pediatric dentists, orthodontists, speech-language therapists, otolaryngologists, and caregivers is important when palatal constriction is associated with speech difficulty, feeding problems, mouth breathing, or sleep-disordered breathing [75,78,79].

Malocclusion

Malocclusion is highly prevalent in individuals with DS and results from a combination of craniofacial growth disturbances, maxillary hypoplasia, delayed tooth eruption, hypodontia, muscular hypotonia, altered tongue posture, mouth breathing, and impaired orofacial function [80-84]. The most frequently reported occlusal abnormalities include anterior open bite, anterior and posterior crossbite, skeletal or dental Class III relationship, reverse overjet, reduced overbite, and dental crowding [80-87]. Anterior open bite is commonly associated with tongue protrusion, low tongue posture, mouth breathing, and abnormal swallowing patterns, whereas posterior crossbite is usually related to transverse maxillary deficiency and a narrow maxillary arch [80,81,87].

Class III malocclusion is particularly characteristic of DS and is often caused by maxillary underdevelopment rather than true mandibular prognathism alone [82,84,87]. Dental crowding may occur when arch dimensions are reduced or when tooth eruption is irregular, even though some individuals may also show spacing because of microdontia or hypodontia [83,86]. These occlusal abnormalities can negatively affect mastication, speech articulation, oral hygiene access, periodontal health, facial esthetics, and quality of life [82-87]. Early orthodontic assessment is therefore recommended to identify developing crossbite, open bite, severe crowding, or skeletal discrepancy and to determine whether interceptive treatment is appropriate.

Management should be individualized and realistic. Interceptive approaches may include monitoring, habit control, myofunctional support, maxillary expansion, correction of functional crossbite, or orthopedic management of Class III tendency when cooperation and medical status permit [88]. Comprehensive orthodontic treatment, including fixed appliances, may be possible in selected patients with DS, but it is often more challenging because of reduced cooperation, oral hygiene limitations, periodontal susceptibility, short or abnormal roots, and increased risk of treatment interruption or complications [89]. Therefore, treatment planning should balance functional benefit, esthetic goals, periodontal risk, airway considerations, and the child’s ability to tolerate long-term orthodontic care [88-90].

Airway and sleep-related problems (OSA)

OSA is highly prevalent in children with DS, with studies reporting rates substantially higher than those observed in the general pediatric population [91-94]. Meta-analytic and polysomnography-based studies have shown that OSA is common across childhood in DS, and moderate-to-severe disease may occur even in young children [91-93]. This increased susceptibility is multifactorial and reflects the combined effects of midface hypoplasia, maxillary restriction, relative macroglossia, adenotonsillar hypertrophy, generalized hypotonia, obesity, glossoptosis, narrow nasopharyngeal dimensions, and increased upper-airway collapsibility during sleep [94-96].

Clinically, OSA may present with habitual snoring, restless or fragmented sleep, witnessed apneas, gasping, mouth breathing, unusual sleep positions, nocturnal sweating, morning fatigue, excessive daytime sleepiness, irritability, behavioral problems, impaired attention, and poor school performance [94-97]. However, symptoms and caregiver reports may correlate poorly with polysomnography findings in children with DS; significant OSA may be present even when typical symptoms are absent or mild [92,95,97]. Untreated OSA can contribute to neurocognitive impairment, behavioral difficulties, growth problems, pulmonary hypertension, cardiovascular strain, metabolic dysfunction, and reduced quality of life [94,96-98].

Because of the high prevalence of OSA and the limited reliability of symptoms alone, routine screening and formal sleep evaluation are essential components of health supervision in children with DS [95,97,98]. Overnight polysomnography remains the diagnostic gold standard and is recommended even in children who appear clinically asymptomatic, particularly during early childhood and whenever symptoms suggest sleep-disordered breathing [95,97,98]. Management should be individualized and may include weight optimization, treatment of allergic rhinitis or nasal obstruction, adenotonsillectomy when adenotonsillar hypertrophy is present, continuous positive airway pressure therapy, orthodontic or craniofacial interventions, and multidisciplinary follow-up [96,99-101]. Residual OSA after adenotonsillectomy is common in DS, so postoperative reassessment and long-term surveillance are important [99-101].

The principal airway manifestations of DS and their implications for dental treatment, sedation, and general anesthesia are summarized in Table 2.

**Table 2:** Airway manifestations of Down syndrome and implications for dental and anesthetic care

Airway manifestation	Clinical features and dental relevance	Recommended dental or anesthetic action
Midface or maxillary hypoplasia and narrow nasopharynx	Reduced upper-airway dimensions may contribute to chronic nasal obstruction, mouth breathing, and obstructive sleep apnea [65-73,94-96].	Ask about nasal obstruction and sleep symptoms, consider otolaryngology or sleep referral, and anticipate reduced airway reserve during sedation.
Relative macroglossia and small oral cavity	The tongue appears large relative to the restricted oral cavity; glossoptosis and airway crowding may occur [57-59,94-96,102-104].	Use careful positioning and suction, recognize the increased risk of obstruction with sedatives, and ensure that airway-rescue equipment and trained personnel are available.
Adenotonsillar hypertrophy	A common contributor to snoring, witnessed apnea, and obstructive sleep apnea [94-101].	Refer for otolaryngology and sleep evaluation when suspected. Residual obstructive sleep apnea after adenotonsillectomy is common and requires reassessment.
Generalized hypotonia and increased airway collapsibility	Reduced pharyngeal tone can impair airway patency and spontaneous ventilation, especially during sedation, anesthesia, and recovery [94-96,102-104].	Titrate sedative medication cautiously, monitor ventilation as well as oxygenation, and be prepared to provide airway support.
Obstructive sleep apnea	Habitual snoring, gasping, restless sleep, unusual sleep positions, daytime fatigue, or behavioral changes may occur; symptoms may underestimate disease severity [91-98].	Screen routinely and refer for polysomnography when indicated. Significant or untreated disease may require hospital-based pediatric anesthesia and extended recovery monitoring.
Smaller tracheal or subglottic caliber	An endotracheal tube predicted by age may be too large, increasing the risk of traumatic intubation or post-extubation stridor [105-107].	Inform the anesthesia team, consider a smaller tube and leak assessment, and document any history of stridor or difficult intubation.
Tracheomalacia, tracheal bronchus, or tracheal/subglottic stenosis	Lower-airway anomalies occur more frequently and may present with recurrent croup, wheeze, respiratory infections, or ventilation difficulty [102-107].	Review previous airway investigations, seek specialist assessment when symptomatic, and use a setting with pediatric difficult-airway expertise for advanced procedures.
Recovery-phase airway vulnerability	Airway obstruction and hypoventilation may occur during emergence and recovery, particularly with obstructive sleep apnea, hypotonia, obesity, or previous airway surgery [108-110].	Provide close postoperative observation and appropriate monitoring, use respiratory-depressant medication cautiously, and delay discharge until airway patency and ventilation are stable.

Dental anesthesia and sedation considerations

Airway Management Difficulties

Airway management during dental sedation and general anesthesia can be challenging in individuals with DS because of characteristic anatomical and physiological airway features [102-106]. Important contributing factors include midface hypoplasia, a relatively small oral cavity, relative macroglossia, adenotonsillar hypertrophy, generalized hypotonia, OSA, and increased upper-airway collapsibility [102-104]. Lower-airway abnormalities, including smaller tracheal caliber, tracheomalacia, tracheal bronchus, and subglottic or tracheal stenosis, are also reported more frequently in children with DS than in controls and may complicate endotracheal intubation or tube selection [104-107].

These airway characteristics may increase the risk of airway obstruction during induction, procedural sedation, emergence, and recovery, particularly in patients with untreated OSA, obesity, hypotonia, or adenotonsillar hypertrophy [102,108]. Mask ventilation may be affected by poor tone, upper-airway obstruction, and craniofacial morphology, while intubation may require careful tube-size selection because children with DS may have a smaller subglottic or tracheal diameter than expected for age [105,106]. Although difficult intubation is not universal in DS, perioperative respiratory adverse events, especially airway obstruction, are reported more frequently than in matched controls [108].

Comprehensive preoperative assessment is therefore essential before dental sedation or general anesthesia. The dentist and anesthesia team should document a history of snoring, witnessed apnea, mouth breathing, previous difficult intubation, post-extubation stridor, recurrent croup, CHD, pulmonary hypertension, respiratory infections, and prior airway surgery [102-104,109,110]. When significant OSA, airway symptoms, complex cardiac disease, or previous anesthetic difficulty is present, treatment should be planned in a hospital-based or specialist setting with pediatric anesthesia expertise. Intraoperative care should include appropriate airway equipment, readiness to manage obstruction or laryngospasm, cautious drug selection, and consideration of smaller endotracheal tubes when intubation is required [108,109]. Close postoperative monitoring is important because airway obstruction and hypoventilation may occur during recovery, especially in children with hypotonia or sleep-disordered breathing [108-110].

Atlantoaxial Instability

Atlantoaxial instability (AAI) is an important musculoskeletal concern in individuals with DS, with radiographic instability reported in approximately 10-20% of affected individuals [111,112]. The condition results from excessive mobility between the atlas (C1) and axis (C2), mainly due to generalized ligamentous laxity, increased laxity of the transverse ligament, hypotonia, and structural abnormalities of the odontoid process or upper cervical vertebrae [112-114]. Although most cases are asymptomatic, a small proportion of individuals may develop symptomatic cervical spinal cord compression, which can result in neck pain, torticollis, gait disturbance, weakness, hyperreflexia, clonus, loss of coordination, sensory changes, or bowel and bladder dysfunction [111-114].

AAI has important implications for dental management, particularly because excessive cervical flexion, extension, or rotation may increase the risk of spinal cord compromise during airway manipulation, endotracheal intubation, patient transfer, or prolonged positioning in susceptible individuals [113-115]. In addition, children with DS have increased upper-airway vulnerability due to craniofacial abnormalities, generalized hypotonia, relative macroglossia, and the high prevalence of OSA. A recent Saudi-affiliated study further demonstrated significant associations between craniofacial characteristics and OSA severity in children with craniofacial syndromes [116]. Therefore, careful preoperative airway assessment and maintenance of a neutral, well-supported head and cervical spine position are recommended throughout dental care. This precaution should be observed not only during sedation, general anesthesia, and airway management, but also during routine awake dental treatment in the dental chair, where unnecessary cervical flexion or extension should be avoided, particularly in children with suspected or confirmed cervical spine instability [2,115,117].

Preoperative assessment should include targeted screening for symptoms suggestive of cervical spine instability or myelopathy, including neck pain, limited neck movement, abnormal gait, new weakness, change in hand function, hyperreflexia, clonus, sensory disturbance, or bowel and bladder changes [113-116]. Routine radiographic screening in asymptomatic children remains controversial, and recent reviews do not support universal imaging in all asymptomatic patients because radiographic findings may correlate poorly with clinical risk [116,117]. However, patients with neurological symptoms, abnormal examination findings, previous cervical spine disease, planned procedures requiring significant neck manipulation, or anticipated difficult airway management should be referred for medical evaluation and appropriate imaging before elective dental treatment under sedation or general anesthesia [113,116,117].

Cardiac considerations

CHD is one of the most important systemic comorbidities in individuals with DS, affecting approximately one-half of patients in large pooled analyses [118]. In Saudi Arabia, recent meta-analytic data suggest an even higher pooled prevalence of CHD among individuals with DS, although estimates vary across studies because of differences in study design, diagnostic methods, and population characteristics [119]. The most frequently reported cardiac defects include atrioventricular septal defect, ventricular septal defect, atrial septal defect, patent ductus arteriosus, and, less commonly, tetralogy of Fallot [118-120]. These conditions may influence dental care because of potential risks related to infective endocarditis, pulmonary hypertension, anticoagulant or cardiac medication use, oxygen desaturation, reduced physiological reserve, and the need for careful planning before sedation or general anesthesia [121,122].

Before invasive dental procedures, clinicians should obtain a detailed cardiac history, including the specific cardiac diagnosis, history of surgical or catheter-based repair, presence of residual defects, cyanosis, pulmonary hypertension, current medications, previous infective endocarditis, and the most recent cardiology follow-up [121-123]. It is also essential to determine whether the patient meets criteria for infective endocarditis prophylaxis. Current guidance does not recommend antibiotic prophylaxis solely because a patient has DS or any form of uncomplicated CHD [121-123]. Prophylaxis is reserved for selected high-risk cardiac conditions, including prosthetic cardiac valves or prosthetic material used for valve repair, previous infective endocarditis, unrepaired cyanotic CHD, repaired CHD with prosthetic material or device during the first six months after repair, repaired CHD with residual defects at or adjacent to a prosthetic patch or device, and cardiac transplant with valvular regurgitation [121-123]. When prophylaxis is indicated, it applies to dental procedures involving manipulation of gingival tissue, manipulation of the periapical region of teeth, or perforation of the oral mucosa [121-123].

Close collaboration among the dentist, pediatrician, and pediatric cardiologist is recommended when the cardiac diagnosis is unclear, the patient has residual lesions or pulmonary hypertension, the child is medically unstable, or dental treatment requires sedation or general anesthesia [121,122]. Preventive dental care is especially important because poor oral hygiene, gingival inflammation, and untreated dental infection may increase the risk of bacteremia from daily activities as well as from dental procedures [121-123]. Therefore, children with DS and CHD should receive early dental referral, individualized caries and periodontal prevention, caregiver education, and timely treatment of oral infection before elective cardiac surgery whenever feasible.

Other oral findings

Beyond the well-recognized dental and craniofacial anomalies, individuals with DS may present with several additional oral and orofacial findings that can affect oral health, function, comfort, and quality of life [124,125]. Fissured tongue is one of the most frequently reported oral mucosal findings and is characterized by multiple grooves or fissures on the dorsal surface of the tongue [124,125]. Although usually benign and asymptomatic, deep fissures may retain food debris and plaque, increasing the need for tongue hygiene and caregiver-assisted oral care [124]. Lip fissuring and angular cheilitis are also commonly observed and may be related to chronic drooling, mouth breathing, lip incompetence, saliva pooling at the oral commissures, and secondary Candida colonization or infection [124-127].

Mouth breathing and excessive drooling are frequent functional concerns in DS and are commonly associated with relative macroglossia, generalized hypotonia, reduced lip closure, impaired oral-motor coordination, and upper-airway obstruction [124,128-131]. Delayed speech development may arise from a combination of cognitive impairment, hearing loss, craniofacial differences, hypotonia, reduced oral-motor precision, and altered sensory feedback [130,131]. Feeding and swallowing difficulties are also common and may involve poor chewing efficiency, food selectivity, prolonged feeding time, coughing during meals, oral-phase dysphagia, pharyngeal dysphagia, aspiration, and silent aspiration [128-131]. These problems may contribute to poor nutrition, respiratory morbidity, caregiver stress, and impaired growth if not recognized and managed early [128-131].

Temporomandibular joint dysfunction may occur in some individuals with DS and may be influenced by ligamentous laxity, muscular hypotonia, malocclusion, parafunctional habits, and bruxism [132,133]. In addition, individuals with DS may be more susceptible to oral Candida carriage and oropharyngeal candidiasis because of immune dysregulation, reduced salivary clearance, mouth breathing, lip lesions, poor oral hygiene, and other systemic or local risk factors [124,126,127]. Therefore, routine dental visits should include inspection of the oral mucosa, lips, tongue, commissures, salivary control, occlusion, oral hygiene, swallowing-related history, and signs of discomfort or functional limitation.

Management should be preventive, individualized, and multidisciplinary. Pediatric dentists should provide early dental-home establishment, caregiver education, oral hygiene reinforcement, fluoride-based prevention, dietary counseling, management of oral mucosal lesions, and timely referral when functional problems are identified [134]. Coordination with pediatricians, otolaryngologists, speech-language therapists, feeding specialists, orthodontists, periodontists, sleep specialists, and anesthesiologists may be required when drooling, dysphagia, sleep-disordered breathing, speech impairment, recurrent candidiasis, severe malocclusion, or the need for sedation or general anesthesia is present [128-136]. Regular follow-up is essential to reduce complications, improve oral comfort and function, and support long-term quality of life in individuals with DS.

General dental recommendations

General dental care for children with DS should be preventive, individualized, family-centered, and coordinated with the medical team. The following recommendations synthesize the practical implications of the reviewed evidence [2,9-13,42-56,88-123,128-136].

Early Dental Care and Risk-Based Review

Establish a dental home early, complete a comprehensive baseline assessment, and determine recall frequency according to caries risk, periodontal status, oral hygiene, cooperation, and medical complexity rather than using a uniform interval.

Updated Medical Assessment

At each visit, review CHD and repair status, pulmonary hypertension, OSA or snoring, airway surgery, recurrent respiratory symptoms, medications, previous anesthesia or intubation problems, and symptoms of cervical spine disease. Obtain a medical, cardiology, sleep, or anesthesia consultation when the diagnosis is unclear or advanced treatment is planned.

Adapted Communication and Behavior Guidance

Use short and predictable appointments, desensitization visits, visual supports, tell-show-do, positive reinforcement, simple language, and active caregiver participation. Treatment goals should reflect the child's communication abilities, cooperation, sensory needs, and tolerance.

Intensive Prevention and Caregiver-Supported Home Care

Recommend caregiver-assisted twice-daily brushing with fluoridated toothpaste, interdental cleaning when feasible, dietary counseling, professional topical fluoride, and fissure sealants according to individual risk. Treat active infection and cavitated lesions without unnecessary delay.

Early and Continuing Periodontal Surveillance

Recording gingival inflammation and periodontal findings from an early age, reinforcing plaque control at every visit, and providing professional debridement or shorter maintenance intervals when needed. Refer for periodontal assessment when pocketing, attachment loss, mobility, or radiographic bone loss is detected.

Monitoring of Eruption and Dental Anomalies

Assess eruption sequence, retained primary teeth, tooth number, enamel defects, and tooth morphology. Use panoramic or other radiographic examination when clinically indicated to distinguish agenesis from delayed eruption or impaction and to support orthodontic, restorative, and prosthetic planning.

Realistic Orthodontic and Functional Planning

Arrange early orthodontic assessment for crossbite, open bite, Class III tendency, maxillary constriction, and severe crowding. Before active treatment, confirm adequate plaque control, periodontal stability, suitable root morphology, medical fitness, and the ability of the child and caregiver to maintain long-term cooperation.

Routine Airway and Sleep Screening

Ask caregivers about snoring, witnessed apnea, gasping, mouth breathing, restless sleep, unusual sleep positions, morning fatigue, and daytime behavioral or attention changes. Refer for otolaryngology or sleep assessment and polysomnography when sleep-disordered breathing is suspected, because symptoms alone may underestimate OSA.

Safe Positioning and Procedural Planning

Maintain the head and neck in a neutral, well-supported position during routine awake restorative care as well as during sedation or general anesthesia, and avoid unnecessary cervical flexion, extension, or rotation. Children with significant OSA, complex airway disease, severe cardiac disease, or previous anesthetic difficulty should be treated in a specialist or hospital setting with pediatric anesthesia and postoperative monitoring capability.

Cardiac Precautions and Antibiotic Prophylaxis 

Confirm the exact cardiac diagnosis, repair status, residual defects, pulmonary hypertension, medications, and cardiology follow-up before invasive procedures. DS alone is not an indication for antibiotic prophylaxis; prophylaxis should be limited to guideline-defined high-risk cardiac conditions and qualifying dental procedures [121-123].

Multidisciplinary Referral

Coordinate care with pediatricians, cardiologists, otolaryngologists, sleep specialists, speech-language therapists, feeding teams, orthodontists, periodontists, and anesthesiologists when airway, swallowing, speech, periodontal, orthodontic, cardiac, or behavioral problems exceed the scope of general dental care.

## Conclusions

Individuals with DS present with diverse oral, dental, craniofacial, periodontal, functional, and airway-related challenges that require early recognition and individualized dental care. Although caries risk may be comparable to or lower than that of the general population, periodontal disease remains a major concern due to early onset, rapid progression, immune dysfunction, and oral hygiene difficulties. Dental management must also account for systemic risks, including OSA, airway anomalies, hypotonia, AAI, and CHD, particularly before sedation or general anesthesia. Antibiotic prophylaxis should be limited to patients with guideline-defined high-risk cardiac conditions and not used for DS alone. Early dental engagement, preventive care, regular surveillance, and multidisciplinary collaboration are essential to improve oral health, procedural safety, function, quality of life, and long-term outcomes in individuals with DS.

## References

[1] Antonarakis SE, Skotko BG, Rafii MS (2020). Down syndrome. Nat Rev Dis Primers.

[2] Bull MJ, Trotter T, Santoro SL (2022). Health supervision for children and adolescents with Down syndrome. Pediatrics.

[3] Niazi MA, al-Mazyad AS, al-Husain MA, al-Mofada SM, al-Zamil FA, Khashoggi TY, al-Eissa YA (1995). Down's syndrome in Saudi Arabia: incidence and cytogenetics. Hum Hered.

[4] Macho V, Palha M, Macedo AP, Ribeiro O, Andrade C (2013). Comparative study between dental caries prevalence of Down syndrome children and their siblings. Spec Care Dentist.

[5] Desai SS (1997). Down syndrome: a review of the literature. Oral Surg Oral Med Oral Pathol Oral Radiol Endod.

[6] Pilcher ES (1998). Dental care for the patient with Down syndrome. Down Syndr Res Pract.

[7] de Moraes ME, de Moraes LC, Dotto GN, Dotto PP, dos Santos LR (2007). Dental anomalies in patients with Down syndrome. Braz Dent J.

[8] Díaz-Quevedo AA, Castillo-Quispe HM, Atoche-Socola KJ, Arriola-Guillén LE (2021). Evaluation of the craniofacial and oral characteristics of individuals with Down syndrome: a review of the literature. J Stomatol Oral Maxillofac Surg.

[9] Hore K, Ali U (2024). Anaesthesia for the child with trisomy 21. BJA Educ.

[10] Botero JE, Rodríguez-Medina C, Amaya-Sánchez S (2024). A comprehensive review of the relationship between oral health and Down syndrome. Curr Oral Health Rep.

[11] Yehia Z, Silbereisen A, Koletsi D, Arabzadehtousi M, Tsilingaridis G, Bostanci N (2024). Efficacy of periodontal treatment modalities in Down syndrome patients: a systematic review and meta-analysis. Evid Based Dent.

[12] Martins M, Mascarenhas P, Evangelista JG, Barahona I, Tavares V (2022). The incidence of dental caries in children with Down syndrome: a systematic review and meta-analysis. Dent J (Basel).

[13] Arroyo-Bote S, Bennasar-Verger C, López-González ÁA (2024). Dental care for patients with Down syndrome: a survey for dentists of the College of the Balearic Islands. J Clin Exp Dent.

[14] Cohen MM, Winer RA (1965). Dental and facial characteristics in Down's syndrome. (Mongolism). Bull Acad Dent Handicap.

[15] Ondarza A, Jara L, Muñoz P (1997). Sequence of eruption of deciduous dentition in a Chilean sample with Down's syndrome. Arch Oral Biol.

[16] Jara L, Ondarza A, Blanco R, Valenzuela C (1993). The sequence of eruption of the permanent dentition in a Chilean sample with Down's syndrome. Arch Oral Biol.

[17] Noda K, Hanaoka S, Watanabe M (2023). Survey of primary tooth eruption status of Down syndrome in Japan: comparison with the Japanese National Survey of Dental Diseases. Pediatr Dent J.

[18] Noda K, Hanaoka S, Watanabe M (2024). Survey of permanent tooth eruption status of Down syndrome in Japan: comparison with the Japanese National Survey of Dental Diseases. Pediatr Dent J.

[19] Gallo C, Pastore I, Beghetto M, Mucignat-Caretta C (2019). Symmetry of dental agenesis in Down syndrome children. J Dent Sci.

[20] Hisamoto N, Watanabe M, Hayashi S (2025). Deciduous teeth eruption, gross motor skills, and feeding in children with Down syndrome: a cross-sectional study. Pediatr Dent J.

[21] Suri S, Tompson BD, Atenafu E (2011). Prevalence and patterns of permanent tooth agenesis in Down syndrome and their association with craniofacial morphology. Angle Orthod.

[22] Palaska PK, Antonarakis GS (2016). Prevalence and patterns of permanent tooth agenesis in individuals with Down syndrome: a meta-analysis. Eur J Oral Sci.

[23] Kalf-Scholte SM, Wijk AV, Mayoral Trias A, Valkenburg C (2024). Patterns of tooth agenesis in individuals with Down syndrome: a secondary analysis using the Tooth Agenesis Code. Spec Care Dentist.

[24] Reuland-Bosma W, Reuland MC, Bronkhorst EM (2010). Patterns of tooth agenesis in patients with Down syndrome in relation to hypothyroidism and congenital heart disease: an aid for treatment planning. Am J Orthod Dentofacial Orthop.

[25] Kumasaka S, Miyagi A, Sakai N, Shindo J, Kashima I (1997). Oligodontia: a radiographic comparison of subjects with Down syndrome and normal subjects. Spec Care Dentist.

[26] Al-Ani AH, Antoun JS, Thomson WM, Merriman TR, Farella M (2017). Hypodontia: an update on its etiology, classification, and clinical management. Biomed Res Int.

[27] Sekerci AE, Cantekin K, Aydinbelge M, Ucar Fİ (2014). Prevalence of dental anomalies in the permanent dentition of children with Down syndrome. J Dent Child (Chic).

[28] Cuoghi OA, Topolski F, Perciliano de Faria L, Occhiena CM, Ferreira ND, Ferlin CR, Rogério de Mendonça M (2016). Prevalence of Dental Anomalies in Permanent Dentition of Brazilian Individuals with Down Syndrome. Open Dent J.

[29] Mayoral-Trias MA, Llopis-Perez J, Puigdollers Pérez A (2016). Comparative study of dental anomalies assessed with panoramic radiographs of Down syndrome and non-Down syndrome patients. Eur J Paediatr Dent.

[30] Anggraini L, Rizal MF, Indiarti IS (2019). Prevalence of dental anomalies in Indonesian individuals with Down syndrome. Pesqui Bras Odontopediatria Clin Integr.

[31] Scott AM, Reed WM, Ajwani S, Parmenter TR (2023). Panoramic radiographs and dental patients with Down syndrome: a scoping review. Spec Care Dentist.

[32] Abeleira MT, Outumuro M, Ramos I, Limeres J, Diniz M, Diz P (2014). Dimensions of central incisors, canines, and first molars in subjects with Down syndrome measured on cone-beam computed tomographs. Am J Orthod Dentofacial Orthop.

[33] Matabuena Rodríguez M, Diz Dios P, Cadarso-Suárez C, Diniz-Freitas M, Outumuro Rial M, Abeleira Pazos MT, Limeres Posse J (2017). Reassessment of fluctuating dental asymmetry in Down syndrome. Sci Rep.

[34] Khurana S, Khalifa AR, Rezallah NN, Lozanoff S, Abdelkarim AZ (2024). Craniofacial and airway morphology in Down syndrome: a cone-beam computed tomography case series evaluation. J Clin Med.

[35] Lobbezoo F, Ahlberg J, Glaros AG (2013). Bruxism defined and graded: an international consensus. J Oral Rehabil.

[36] Lobbezoo F, Ahlberg J, Raphael KG (2018). International consensus on the assessment of bruxism: report of a work in progress. J Oral Rehabil.

[37] Luconi E, Togni L, Mascitti M (2021). Bruxism in children and adolescents with Down syndrome: a comprehensive review. Medicina (Kaunas).

[38] Alam MK, Alsharari AH, Shayeb MA, Elfadil S, Cervino G, Minervini G (2023). Prevalence of bruxism in Down syndrome patients: a systematic review and meta-analysis. J Oral Rehabil.

[39] Sobiech L, Dąbkowska I, Bekiesz W, Turżańska K, Blicharski T, Sarna-Boś K (2024). Bruxism in a child with trisomy 21 (Down syndrome): case report. J Clin Med.

[40] Carra MC, Bruni O, Huynh N (2012). Topical review: sleep bruxism, headaches, and sleep-disordered breathing in children and adolescents. J Orofac Pain.

[41] Huynh N, Fabbro CD (2024). Sleep bruxism in children and adolescents-a scoping review. J Oral Rehabil.

[42] Rondón-Avalo S, Rodríguez-Medina C, Botero JE (2024). Association of Down syndrome with periodontal diseases: systematic review and meta-analysis. Spec Care Dentist.

[43] Scalioni FA, Carrada CF, Martins CC, Ribeiro RA, Paiva SM (2018). Periodontal disease in patients with Down syndrome: a systematic review. J Am Dent Assoc.

[44] Ghaffarpour M, Karami-Zarandi M, Rahdar HA, Feyisa SG, Taki E (2024). Periodontal disease in Down syndrome: predisposing factors and potential non-surgical therapeutic approaches. J Clin Lab Anal.

[45] Vergier V, Collignon AM, Gosset M (2025). Periodontal diseases in Down syndrome during childhood: a scoping review. BMC Oral Health.

[46] Contaldo M, Lucchese A, Romano A, Della Vella F, Di Stasio D, Serpico R, Petruzzi M (2021). Oral microbiota features in subjects with Down syndrome and periodontal diseases: a systematic review. Int J Mol Sci.

[47] Huggard D, Doherty DG, Molloy EJ (2020). Immune dysregulation in children with Down syndrome. Front Pediatr.

[48] Izumi Y, Sugiyama S, Shinozuka O, Yamazaki T, Ohyama T, Ishikawa I (1989). Defective neutrophil chemotaxis in Down's syndrome patients and its relationship to periodontal destruction. J Periodontol.

[49] Amano A, Murakami J, Akiyama S (2008). Etiologic factors of early-onset periodontal disease in Down syndrome. Jpn Dent Sci Rev.

[50] Moreira MJ, Schwertner C, Jardim JJ, Hashizume LN (2016). Dental caries in individuals with Down syndrome: a systematic review. Int J Paediatr Dent.

[51] Deps TD, Angelo GL, Martins CC, Paiva SM, Pordeus IA, Borges-Oliveira AC (2015). Association between dental caries and Down syndrome: a systematic review and meta-analysis. PLoS One.

[52] Silva MC, Lyra MC, Almeida HC, Alencar Filho AV, Heimer MV, Rosenblatt A (2020). Caries experience in children and adolescents with Down Syndrome: a systematic review and meta-analysis. Arch Oral Biol.

[53] Singh V, Arora R, Bhayya D, Singh D, Sarvaiya B, Mehta D (2015). Comparison of relationship between salivary electrolyte levels and dental caries in children with Down syndrome. J Nat Sci Biol Med.

[54] Mathias MF, Simionato MR, Guaré RO (2011). Some factors associated with dental caries in the primary dentition of children with Down syndrome. Eur J Paediatr Dent.

[55] Gufran K, Alqutaym OS, Alqahtani AA, Alqarni AM, Hattan EA, Alqahtani RO (2019). Prevalence of dental caries and periodontal status among the Down syndrome population in Riyadh City. J Pharm Bioallied Sci.

[56] Stein Duker LI, Richter M, Lane CJ, Polido JC, Cermak SA (2020). Oral care experiences and challenges for children with Down syndrome: reports from caregivers. Pediatr Dent.

[57] Guimaraes CV, Donnelly LF, Shott SR, Amin RS, Kalra M (2008). Relative rather than absolute macroglossia in patients with Down syndrome: implications for treatment of obstructive sleep apnea. Pediatr Radiol.

[58] Shukla D, Bablani D, Chowdhry A, Thapar R, Gupta P, Mishra S (2014). Dentofacial and cranial changes in Down syndrome. Osong Public Health Res Perspect.

[59] Al-Shawaf R, Al-Faleh W (2011). Craniofacial characteristics in Saudi Down syndrome. King Saud Univ J Dent Sci.

[60] Kent RD, Vorperian HK (2013). Speech impairment in Down syndrome: a review. J Speech Lang Hear Res.

[61] Cañizares-Prado S, Molina-López J, Moya MT, Planells E (2022). Oral function and eating habit problems in people with Down syndrome. Int J Environ Res Public Health.

[62] Hielscher L, Irvine K, Ludlow AK, Rogers S, Mengoni SE (2023). A scoping review of the complementary feeding practices and early eating experiences of children with Down syndrome. J Pediatr Psychol.

[63] Franceschetti S, Tofani M, Mazzafoglia S (2024). Assessment and rehabilitation intervention of feeding and swallowing skills in children with Down syndrome using the Global Intensive Feeding Therapy (GIFT). Children (Basel).

[64] Javed F, Akram Z, Barillas AP, Kellesarian SV, Ahmed HB, Khan J, Almas K (2018). Outcome of orthodontic palatal plate therapy for orofacial dysfunction in children with Down syndrome: a systematic review. Orthod Craniofac Res.

[65] Suri S, Tompson BD, Cornfoot L (2010). Cranial base, maxillary and mandibular morphology in Down syndrome. Angle Orthod.

[66] Korayem MA, AlKofide EA (2014). Characteristics of Down syndrome subjects in a Saudi sample. Angle Orthod.

[67] Vicente A, Bravo-González LA, López-Romero A, Muñoz CS, Sánchez-Meca J (2020). Craniofacial morphology in Down syndrome: a systematic review and meta-analysis. Sci Rep.

[68] Allareddy V, Ching N, Macklin EA (2016). Craniofacial features as assessed by lateral cephalometric measurements in children with Down syndrome. Prog Orthod.

[69] Bierley K, Antonarakis GS (2023). Lateral cephalometric characteristics in individuals with Down syndrome compared to non-syndromic controls: a meta-analysis. J Stomatol Oral Maxillofac Surg.

[70] Takizawa H, Takahashi M, Maki K (2022). Three-dimensional assessment of craniofacial features in patients with Down syndrome during the mixed dentition period: a case-control study. Cleft Palate Craniofac J.

[71] Doriguêtto PV, Carrada CF, Scalioni FA, Abreu LG, Devito KL, Paiva SM, Ribeiro RA (2019). Malocclusion in children and adolescents with Down syndrome: a systematic review and meta-analysis. Int J Paediatr Dent.

[72] Simpson R, Oyekan AA, Ehsan Z, Ingram DG (2018). Obstructive sleep apnea in patients with Down syndrome: current perspectives. Nat Sci Sleep.

[73] Maris M, Verhulst S, Wojciechowski M, Van de Heyning P, Boudewyns A (2016). Prevalence of obstructive sleep apnea in children with Down syndrome. Sleep.

[74] Skrinjarić T, Glavina D, Jukić J (2004). Palatal and dental arch morphology in Down syndrome. Coll Antropol.

[75] Bhagyalakshmi G, Renukarya AJ, Rajangam S (2007). Metric analysis of the hard palate in children with Down syndrome: a comparative study. Downs Syndr Res Pract.

[76] Klingel D, Hohoff A, Kwiecien R, Wiechmann D, Stamm T (2017). Growth of the hard palate in infants with Down syndrome compared with healthy infants-a retrospective case control study. PLoS One.

[77] Ghaith B, Al Halabi M, Khamis AH, Kowash M (2019). Oral health status among children with Down syndrome in Dubai, United Arab Emirates. J Int Soc Prev Community Dent.

[78] Alkawari H (2021). Down syndrome children, malocclusion characteristics and the need for orthodontic treatment (IOTN): a cross-sectional study. Children (Basel).

[79] Möhlhenrich SC, Schmidt P, Chhatwani S (2023). Orofacial findings and orthodontic treatment conditions in patients with down syndrome - a retrospective investigation. Head Face Med.

[80] Oliveira AC, Paiva SM, Campos MR, Czeresnia D (2008). Factors associated with malocclusions in children and adolescents with Down syndrome. Am J Orthod Dentofacial Orthop.

[81] Oliveira AC, Pordeus IA, Torres CS, Martins MT, Paiva SM (2010). Feeding and nonnutritive sucking habits and prevalence of open bite and crossbite in children/adolescents with Down syndrome. Angle Orthod.

[82] Bauer D, Evans CA, Begole EA, Salzmann L (2012). Severity of occlusal disharmonies in Down syndrome. Int J Dent.

[83] Abdul Rahim FS, Mohamed AM, Nor MM, Saub R (2014). Malocclusion and orthodontic treatment need evaluated among subjects with Down syndrome using the Dental Aesthetic Index (DAI). Angle Orthod.

[84] Marques LS, Alcântara CE, Pereira LJ, Ramos-Jorge ML (2015). Down syndrome: a risk factor for malocclusion severity?. Braz Oral Res.

[85] Andersson EM, Axelsson S, Katsaris KP (2016). Malocclusion and the need for orthodontic treatment in 8-year-old children with Down syndrome: a cross-sectional population-based study. Spec Care Dentist.

[86] Syed Mohamed AM, Wei TZ, Sean CJ, Rosli TI (2023). Comparison of the malocclusion and orthodontic treatment needs of Down syndrome and non-syndromic subjects by using the dental aesthetics index. Spec Care Dentist.

[87] Alessandri-Bonetti A, Guglielmi F, Mollo A, Sangalli L, Gallenzi P (2023). Prevalence of malocclusions in Down syndrome population: a cross-sectional study. Medicina (Kaunas).

[88] Outumuro M, Abeleira MT, Caamaño F, Limeres J, Suarez D, Diz P, Tomás I (2010). Maxillary expansion therapy in children with Down syndrome. Pediatr Dent.

[89] Abeleira MT, Pazos E, Limeres J, Outumuro M, Diniz M, Diz P (2016). Fixed multibracket dental therapy has challenges but can be successfully performed in young persons with Down syndrome. Disabil Rehabil.

[90] Pithon MM, Tanaka OM (2023). Treatment of Class II malocclusion with anterior open bite and posterior crossbite with the aid of mini-implants in a patient with Down syndrome: clinical case report. Spec Care Dentist.

[91] Lee CF, Lee CH, Hsueh WY, Lin MT, Kang KT (2018). Prevalence of obstructive sleep apnea in children with Down syndrome: a meta-analysis. J Clin Sleep Med.

[92] Nerfeldt P, Sundelin A (2020). Obstructive sleep apnea in children with down syndrome - prevalence and evaluation of surgical treatment. Int J Pediatr Otorhinolaryngol.

[93] Al-Naimi AR, Abu-Hasan M, Belavendra A, Janahi I (2024). Prevalence, severity, and treatment of obstructive sleep apnea in children with Down syndrome: a retrospective study. Cureus.

[94] Hanna N, Hanna Y, Blinder H, Bokhaut J, Katz SL (2022). Predictors of sleep disordered breathing in children with Down syndrome: a systematic review and meta-analysis. Eur Respir Rev.

[95] Hizal M, Sucu A, Murat E (2021). Obstructive sleep apnea in children with Down syndrome. J Clin Sleep Med.

[96] Fauroux B, Amaddeo A, Khirani S (2024). Early detection and treatment of obstructive sleep apnoea in infants with Down syndrome: a prospective cohort study. Lancet Reg Health Eur.

[97] Knollman PD, Heubi CH, Meinzen-Derr J, Smith DF, Shott SR, Wiley S, Ishman SL (2019). Adherence to guidelines for screening polysomnography in children with Down syndrome. Otolaryngol Head Neck Surg.

[98] Matarese CA, Slattery WH, Koltai PJ (2023). The impact of obstructive sleep apnea screening guidelines in children with Down syndrome. Int J Pediatr Otorhinolaryngol.

[99] Li Y, Lu Z, Wang Y (2024). Efficacy and safety of adenotonsillectomy in the treatment of obstructive sleep apnea in children with Down syndrome: a systematic review and meta-analysis. Int J Pediatr Otorhinolaryngol.

[100] Tanner S, Collaro A, Chawla J (2023). The management of residual OSA post-adenotonsillectomy in children with Down syndrome: the experience of a large tertiary sleep service. Sleep Med.

[101] Ehsan Z, Ishman SL, Soghier I (2024). Management of persistent, post-adenotonsillectomy obstructive sleep apnea in children: an Official American Thoracic Society Clinical Practice Guideline. Am J Respir Crit Care Med.

[102] Craven VE, Daw WJ, Wan JW, Elphick HE (2025). Respiratory and airway disorders in children with Down syndrome: a review of the clinical challenges and management. Front Pediatr.

[103] Hamilton J, Yaneza MM, Clement WA, Kubba H (2016). The prevalence of airway problems in children with Down's syndrome. Int J Pediatr Otorhinolaryngol.

[104] De Lausnay M, Verhulst S, Boel L, Wojciechowski M, Boudewyns A, Van Hoorenbeeck K (2020). The prevalence of lower airway anomalies in children with Down syndrome compared to controls. Pediatr Pulmonol.

[105] Graber TJ, Baskin PL, Soria C, Greenberg M, Gabriel RA, Brzenski A (2021). An assessment of perioperative respiratory adverse events and difficult intubation in pediatric patients with trisomy 21. Paediatr Anaesth.

[106] Fockens MM, Hölscher M, Limpens J, Dikkers FG (2021). Tracheal anomalies associated with Down syndrome: a systematic review. Pediatr Pulmonol.

[107] Shott SR (2000). Down syndrome: analysis of airway size and a guide for appropriate intubation. Laryngoscope.

[108] Ujita T, Yamamoto T, Tanaka Y, Kurata S, Seo K (2024). Tracheal stenosis detected during endotracheal intubation in a patient with Down syndrome. Anesth Prog.

[109] Tsao M, Yanko F, Cheon E (2025). Perioperative complications in children with Down syndrome: a single-center retrospective analysis—original clinical research report. J Clin Med.

[110] Coté CJ, Wilson S (2025). Guidelines for monitoring and management of pediatric patients before, during, and after sedation for diagnostic and therapeutic procedures. Pediatr Dent.

[111] Pueschel SM, Scola FH (1987). Atlantoaxial instability in individuals with Down syndrome: epidemiologic, radiographic, and clinical studies. Pediatrics.

[112] Ali FE, Al-Bustan MA, Al-Busairi WA, Al-Mulla FA, Esbaita EY (2006). Cervical spine abnormalities associated with Down syndrome. Int Orthop.

[113] Hata T, Todd MM (2005). Cervical spine considerations when anesthetizing patients with Down syndrome. Anesthesiology.

[114] Mik G, Gholve PA, Scher DM, Widmann RF, Green DW (2008). Down syndrome: orthopedic issues. Curr Opin Pediatr.

[115] Litman RS, Zerngast BA, Perkins FM (1995). Preoperative evaluation of the cervical spine in children with trisomy-21: results of a questionnaire study. Paediatr Anaesth.

[116] Alsaeed S, Huynh N, Wensley D (2023). Orthodontic and facial characteristics of craniofacial syndromic children with obstructive sleep apnea. Diagnostics (Basel).

[117] Yang J, Zheng X, He Y, Cao D, Zhang X, Chen Y, Tu Y (2026). A narrative review of dental general anesthesia in children with special health care needs. Int Dent J.

[118] Bouchard M, Bauer JM, Bompadre V, Krengel WF 3rd (2019). An updated algorithm for radiographic screening of upper cervical instability in patients with Down syndrome. Spine Deform.

[119] Cattarinussi L, Bregou A, Newman CJ, Merckaert SR (2025). Radiological screening of atlantoaxial instability in children with Trisomy 21: a systematic review. Children (Basel).

[120] Xu CX, Chen L, Cheng Y, Du Y (2025). Prevalence of congenital heart defects in people with Down syndrome: a systematic review and meta-analysis. J Epidemiol Community Health.

[121] Sharaf R, Garout W, Sharaf R (2022). Prevalence of congenital heart defects in individuals with Down syndrome in Saudi Arabia: a systematic review and meta-analysis. Cureus.

[122] Freeman SB, Taft LF, Dooley KJ (1998). Population-based study of congenital heart defects in Down syndrome. Am J Med Genet.

[123] Wilson WR, Gewitz M, Lockhart PB (2021). Prevention of viridans group streptococcal infective endocarditis: a scientific statement from the American Heart Association. Circulation.

[124] (2025). Antibiotic Prophylaxis for Dental Patients at Risk for Infection. Pediatric Dentistry.

[125] Thornhill M, Prendergast B, Dayer M, Frisby A, Lockhart P, Baddour LM (2024). Prevention of infective endocarditis in at-risk patients: how should dentists proceed in 2024?. Br Dent J.

[126] Contaldo M, Santoro R, Romano A (2021). Oral manifestations in children and young adults with Down syndrome: a systematic review of the literature. Appl Sci.

[127] Al-Maweri SA, Tarakji B, Al-Sufyani GA, Al-Shamiri HM, Gazal G (2015). Lip and oral lesions in children with Down syndrome. a controlled study. J Clin Exp Dent.

[128] Mohiddin G, Narayanaswamy AB, Masthan KM, Nagarajan A, Panda A, Behura SS (2015). Oral Candidal and Streptococcal carriage in Down syndrome patients. J Nat Sci Biol Med.

[129] Komatsu T, Watanabe K, Hamada N, Helmerhorst E, Oppenheim F, Lee MC (2021). Association between antimicrobial peptide histatin 5 levels and oral Candida infection in Down syndrome. Antibiotics (Basel).

[130] Jackson A, Maybee J, Moran MK, Wolter-Warmerdam K, Hickey F (2016). Clinical characteristics of dysphagia in children with Down syndrome. Dysphagia.

[131] Anil MA, Shabnam S, Narayanan S (2019). Feeding and swallowing difficulties in children with Down syndrome. J Intellect Disabil Res.

[132] Serel Arslan S (2022). Swallowing-related problems of toddlers with Down syndrome. J Dev Phys Disabil.

[133] Madhavan A, Lam L, Etter NM, Wilkinson KM (2023). A biophysiological framework exploring factors affecting speech and swallowing in clinical populations: focus on individuals with Down syndrome. Front Psychol.

[134] Salazar AP, Nery JC, Donini L, Nora VP, Peralles RN (2016). Temporomandibular joint evaluation in subjects with Down syndrome. Int Med Rev Down Syndr.

[135] Sena LD, Palma LF, Marques SR, Sanches ML, de Moraes LO (2020). A home-based multidisciplinary programme for Down syndrome adults with muscular temporomandibular disorder. J Oral Rehabil.

[136] (2026). Management of Dental Patients with Special Health Care Needs. Pediatric Dentistry.

