# Correction: Oral Health and Dental Management of Children With Down Syndrome: A Comprehensive Review

**DOI:** 10.7759/cureus.c479

**Published:** 2026-09-11

**Authors:** Abdullah S Alshamrani, Ghada Alshamrani, Abdulaziz Al-Shamrani, Mosleh Al-Shamrani

**Affiliations:** 1 Department of Pediatrics, Riyadh Armed Forces Hospital, Riyadh, SAU; 2 College of Dentistry, Riyadh Elm University, Riyadh, SAU; 3 Department of Pediatric Dentistry and Orthodontics, College of Dentistry, King Saud University, Riyadh, SAU

This article has been corrected to add second, third and fourth authors, who were originally omitted from the original submission and subsequent publication. In addition, acknowledgements have been added at the request of the authors.

